# Workplace incivility as a risk factor for workplace bullying and psychological well-being: a longitudinal study of targets and bystanders in a sample of swedish engineers

**DOI:** 10.1186/s40359-022-00996-1

**Published:** 2022-12-12

**Authors:** Kristoffer Holm, Eva Torkelson, Martin Bäckström

**Affiliations:** 1 Centre for Work Life and Evaluation Studies, Department of Urban Studies, Malmö University, 205 06 Malmö, Sweden; 2 Department of Psychology, Lund University, Lund, Sweden

**Keywords:** Workplace incivility, Workplace bullying, Psychological well-being, Target, Bystander

## Abstract

**Background:**

The aim of the present study is to explore whether (experienced and witnessed) workplace incivility is a risk factor for (experienced and witnessed) workplace bullying. An additional aim is to explore whether experienced workplace incivility is associated with psychological well-being above and beyond the influence of (experienced and witnessed) workplace bullying on well-being.

**Methods:**

A survey was distributed via e-mail to a panel of Swedish engineers. The survey was administered at three time points over one year. In total, *N* = 1005 engineers responded to the survey. Of these, *N* = 341 responded to more than one survey, providing longitudinal data. *N* = 111 responded to all three surveys.

**Results:**

The results showed that the likelihood of being targeted by workplace bullying was higher for those who had previously experienced incivility, even when taking previous bullying exposure into account. There was also partial support for a higher likelihood of witnessing bullying at a later time point for those that had previously witnessed incivility. Additionally, the results showed that experienced workplace incivility was negatively related to psychological well-being over time, even when controlling for previous levels of experienced and witnessed workplace bullying and well-being. However, this result was only found over one of the two time lags.

**Conclusion:**

The findings of the present study suggests that workplace incivility can be a risk factor for future bullying. In addition, the findings suggest that experienced workplace incivility exerts a unique negative effect on psychological well-being, even when accounting for exposure to workplace bullying.

## Introduction

In the most recent iteration of the European Working Conditions Survey, 16% of EU workers reported being exposed to adverse social behavior in the workplace, such as bullying and harassment, over the past month [1]. This demonstrates that workplace mistreatment remains a pervasive work environment issue in the European Union, affecting a large amount of workers every year. Being exposed to mistreatment at work is a well known occupational hazard with clear documented negative outcomes [1]. For instance, the consequences of being subjected to workplace bullying include depression, anxiety and stress-related complaints [2], post traumatic stress [3], suicidal ideation [4, 5], cardiovascular disease and type II diabetes [6, 7], sickness absence [8, 9], and many other health and work-related outcomes [10].

Workplace bullying is commonly defined as harassing, offending, or socially excluding someone repeatedly over time, where the target is in an inferior position, and systematically targeted by negative social acts [11]. Bullying can therefore be considered a severe form of mistreatment. However, over the past decades, research has begun to focus on whether workplace mistreatment with a lower intensity also could be an occupational hazard for employees. About two decades ago, Andersson and Pearson [12] published a seminal paper on workplace incivility, which they defined as “low intensity deviant behavior, with ambiguous intent to harm the target” [12, p. 457]. Overall, workplace incivility was described as a subtle form of interpersonal mistreatment in the workplace, consisting of rude behaviors such as derogatory or condescending comments, interrupting others, not listening, or having a dismissive body language [13]. As for incivility, it is however not clear whether the behavior is intentional (hence the ambiguity), and it can concern single incidents rather than systematic abuse toward a specific target in an inferior power position.

Since 1999, extensive research has been conducted on the outcomes of workplace incivility demonstrating clear detrimental outcomes for both targets and witnesses [14]. This indicates that even low intensity mistreatment with unclear intentionality can be harmful to employees’ occupational health, and thus worthy of further attention. Moreover, workplace incivility was also described to, despite its low intensity, be at risk of developing into increasingly intense and aggressive behaviors if left unaddressed [12]. This suggests that workplace incivility can be a precursor to workplace bullying, although this has not yet been explored empirically. Specifically, incivility may run the risk of developing into bullying over time, when rude and condescending behavior from coworkers or supervisors in the workplace become systematic, repetitive, and intentional.

In addition to this, Hershcovis [15] has criticized that these factors, despite being central distinguishing features between incivility and bullying, often have been unmeasured in studies on each construct. Instead, it has been argued that the constructs generally have been indistinguishable from oneanother in research studies, which complicates inferences relating to each type of mistreatment [15]. This can be especially problematic as occurences of workplace incivility and bullying are likely to covary. There is therefore a gap in knowledge about the actual unique consequences of workplace incivility, when taking into account that individuals that report incivility also may have been exposed to other unmeasured types of more severe mistreatment.

To address these knowledge gaps, the aim of the present study is to explore whether (experienced and witnessed) workplace incivility is a risk factor for (experienced and witnessed) workplace bullying. An additional aim is to explore whether experienced workplace incivility is associated with psychological well-being above and beyond the influence of (experienced and witnessed) workplace bullying on well-being.

### Workplace incivility as a risk factor for workplace bullying

Workplace mistreatment has often been described as an escalating process in organizations, which may start with mistreatment of a lower intensity, that gradually and over time evolves into more severe forms of mistreatment [11, 16]. This is particularly evident when considering how workplace incivility and workplace bullying typically have been defined. For instance, workplace incivility has been defined as low intensity aggression where the perpetrators’ intentions are ambiguous to interpret [12]. Workplace bullying, on the other hand, consists of persistent negative acts [11]. The high intensity of overt harassment also makes bullying behaviors unambiguous to interpret [17]. However, by following these definitions, incivility that is repeated over time, and that results in a power imbalance, would theoretically escalate into bullying [18].

Consistent with this line of reasoning, Andersson and Pearson [12] described that workplace incivility can escalate through a negative spiral of reciprocal ‘tit-for-tat’ exchanges. In such cases, two parties may initially exchange adverse behaviors of low intensity, that subsequently become increasingly more intentional and intense in a downward spiraling pattern, as the stakes and frustration rises on both sides. Andersson and Pearson [12] argued that adverse social behavior eventually reaches a tipping point, where it no longer is seen as unintentional or ambiguous, and instead perceived as intentional aggression. Ultimately, it has been suggested that workplace incivility can contribute to eroding norms for respect in the workplace, and result in a culture of incivility where interpersonal rudeness spreads throughout the organization [12, 17, 19]. It is possible that the spread of workplace incivility in an organization also could result in the occurrence of mistreatment with an increased intensity.

Likewise, workplace bullying has been described as an escalating process, which gradually worsens over time [20]. The bullying process has been described to start with an initial work-related conflict which results in tension. In a second step, the conflict shifts from work-oriented to person-oriented, and more overt hostility is introduced. In the third, and final step, targets are increasingly dehumanized, perceived as deserving of mistreatment, and subjected to severe negative treatment [11]. Empirically, such an escalating pattern has been supported based on qualitative interviews with victims of bullying [16]. In more recent work, it has also been demonstrated that individuals currently exposed to incivility, individuals at risk of bullying, and individuals exposed to severe bullying, can be differentiated based on the frequency and amount of negative acts they report [21, 22]. Consistent with theoretical escalation models, work-related bullying acts were most commonly observed in the initial phases, whereas person-oriented bullying acts to a higher extent characterized severe bullying cases [21]. However, it has also been noted that bullying can occur in the form of ‘predatory bullying’ [23]. In such cases, there is no prior conflict between the target and perpetrator that escalates into bullying. Instead, bullying could emerge as a consequence of prejudice, being a scapegoat, or as a demonstration of power by the perpetrator [23]. Even though predatory expressions of workplace bullying also have been discussed in the literature, the focus of the present study is on the escalation process of bullying.

Although theoretical models of workplace mistreatment suggest that it can be an escalating process, few studies have examined this more specifically. Zapf and Gross [16] analyzed interview data from 20 individuals, and the studies by Rosander and Blomberg [21] as well as Nixon et al. [22] explored cross-sectional between-subject differences in mistreatment exposure, rather than within-individual change over time. A knowledge gap therefore remains about whether workplace mistreatment of a lower intensity over time can develop into more severe harassment. One prerequisite for escalation is that there is a relationship between low and high intensity mistreatment over time. In the present study, we explore whether individuals that have experienced incivility are at higher risk of being exposed to bullying over time. Additionally, we explore whether witnesses to incivility are more prone to witness workplace bullying over time, to explore whether incivility can be a precursor to more severe harassment. By studying both targets and bystanders, we comprehensively investigate whether incivility is a risk factor for the future presence of bullying in the workplace. We hypothesize:

#### H1

Experienced workplace incivility is significantly positively related to self-reports of bullying exposure over time (from t1 to t2, and from t2 to t3).

#### H2

Witnessed workplace incivility is significantly positively related to self-reports of witnessed bullying over time (from t1 to t2, and from t2 to t3).

### The relationship between workplace incivility and psychological well-being

Experienced workplace incivility has been related to lower levels of health and well-being in several studies [24–26]. There is therefore substantial support for the notion that workplace incivility has negative health effects. Although it is a stressor of low intensity, it has previously been argued that frequent exposure to minor stressful events can be stressful, and cause strain over time [27]. In this way, workplace incivility has been conceptualized as a ‘daily hassle’ [28], that is frustrating to employees and detrimental to their health and well-being as the stress results in allostatic load.

Unsurprisingly, high intensity mistreatment such as workplace bullying has also been shown to have a clear negative relationship with health and well-being in several studies (see Mikkelsen et al. [29]; or Nielsen and Einarsen [10] for two recent reviews). Nevertheless, workplace incivility and workplace bullying have in most studies been assessed separately. Consequently, it is possible that instruments that have been designed to measure workplace incivility also inadvertently may tap into other, more severe mistreatment constructs, such as workplace bullying. It has been pointed out that the items of scales frequently used to measure each construct (i.e. the workplace incivility scale, (WIS) [28]; and the Negative Acts Questionnaire-Revised, (NAQ-R), [30]), have a large degree of content overlap [15]. In part, this creates a credibility problem, as it is difficult to assess whether studies that have found a significant relationship between experienced incivility and well-being have discovered such a relationship due to the unique impact of incivility on well-being, or due to inadvertently measuring exposure to more severe mistreatment. In other words, it is possible that the relationship between incivility and well-being may be confounded by an underlying unobserved variable in these studies, namely workplace bullying. Conversely, the opposite may also be true. The association between workplace bullying and well-being that has been found in several studies, where the NAQ-R has been used, could be confounded by workplace incivility, as the scale may tap into that construct as well. It is therefore important to include workplace incivility and bullying in the same model in order to test the specific influence of each factor on well-being. Unless the possible negative effect that bullying may have on well-being is accounted for, it will be difficult to draw inferences about a relationship between workplace incivility and well-being.

In a recent meta-analysis, incivility was found to incrementally predict several negative outcomes when controlling for constructs such as aggressive behavior, ostracism, undermining, sexual harassment, and abusive supervision [31]. However, they found that incivility had no incremental contribution in the prediction of well-being after controlling for sexual harassment, undermining, ostracism, and abusive supervision [31]. But in a supplementary sample collected by the researchers, incivility did show an incremental prediction of well-being (and several other constructs) beyond the impact of all the aforementioned factors [31]. Nevertheless, the study did not explicitly control for the impact of workplace bullying, a construct that most frequently has been compared to incivility [32]. Thus, a knowledge gap still remains in regard to whether incivility incrementally predicts well-being over workplace bullying. We therefore intend to build on the findings of Yao et al. [31], and test if there is a significant relationship between experienced workplace incivility and well-being, when having controlled for the impact that workplace bullying has on well-being. We also extend the findings by exploring this prediction over time, as a central component in the allostatic load hypothesis is that strain accumulates over time.

In this case, we intend to explore the impact on psychological well-being specifically. Low intensity mistreatment has been shown to be a less pronounced predictor of physical well-being [15]. Conversely, incivility was equally strongly related to psychological well-being as bullying was in a meta-analysis [15]. This is consistent with Lim et al. [25], who found that experienced workplace incivility was directly negatively related to mental health, and only indirectly related to physical health via mental health. This makes psychological well-being a particularly interesting factor when attempting to examine the unique contribution of incivility, above and beyond bullying. Taken together, testing whether incivility contributes unique variance over bullying is a key tenet for the construct validity of workplace incivility, and necessary in order to understand true ramifications of low intensity mistreatment in the workplace. Based on Cortina et al.’s [28] reasoning about workplace incivility as a daily hassle, we hypothesize that:

#### H3

Experienced workplace incivility is significantly negatively related to psychological well-being over time, above and beyond the contribution of workplace bullying in the same model (from t1 to t2, and t2 to t3).

In this case, we do not present any hypothesis about witnessed incivility, as previous research based on the same panel of participants has demonstrated that there is no significant relationship between witnessed incivility and well-being over time [33]. We do however include witnessed incivility in the models testing H3, as experienced incivility and witnessed incivility have been shown to be highly correlated [24, 26]. In this way we obtain more precise parameter estimates and conduct a more stringent test of the present hypothesis.

## Method

### Participants and procedure

A large worker’s union organizing engineers in Sweden was contacted about the study and asked to distribute a survey to their members at three occasions over one year. The rationale for involving engineers in this study was the result of a collaboration between the engineering union and the research team. Therefore, the study is based on convenience. Specifically, sampling from a union has some benefits, as unions have members working in different workplaces across the labor market, this sampling procedure could reduce bias associated with studying a particular workplace or organization. Therefore, the research team sought contact with a union for the study, which in this case resulted in a collaboration with the engineering union. The union was first asked to randomly draw a pool of about 5000 individuals from their membership registry. The survey was then sent via email together with a cover letter about the study to the pool of potential participants at all three occasions. One reminder was sent out at each occasion after the first email. No incentives, rewards, or payments were offered to the participants for participating in the study. The time lag between assessments was roughly six months. Participants’ responses were matched over time by a unique identifier code for each participant. All study variables were measured at all three occasions.

In total, *N* = 1005 (622 male, 380 female, 3 did not report sex) individuals responded to the survey at some point. The mean age of these participants was 45.0 years (*SD* = 10.1), and the average tenure was 8.6 years (*SD* = 8.6). Of the respondents, *N* = 341 individuals responded to at least two surveys, whereas 664 respondents only responded at one occasion. *N* = 111 responded to all three surveys. The survey was initially sent out to 5073 individuals at time 1, 4878 at time 2, and 4630 at time 3. At time 1, 517 (10.2%) responded, at time 2, 498 (10.2%) responded, and at time 3, 490 (10.6%) responded. The overall response rate to the survey was 19.8%. The completion rates, defined as the total number of submitted responses divided by the total number of survey links opened, were 82.3%, 77.4% and 77.8% for time 1, 2 and 3 respectively.

### Measures

#### Demographic data

Questions concerning sex of the participant, age, as well as tenure at the current workplace were included in the survey. Participants also reported whether they had a supervisor position or not, as well as their occupational category. At the time 2 and time 3 surveys, one question was included to assess whether the participants had changed workplace since the last measurement occasion, if they had participated in a previous survey. Respondents that reported having changed workplace over the course of the study were subsequently removed from all analyses (*N* = 23), in order to reduce the risk of drawing inferences about possible bullying exposure at new workplaces, unrelated to the predictor at the previous time point.

#### Workplace incivility (experienced and witnessed)

A Swedish translation [34], of The Workplace Incivility Scale [28] was used to measure experienced incivility. A modified version of the WIS, where stems are changed to assess witnessed rather than experienced behavior, was used to measure witnessed incivility. The scales each consist of 7 aggregated items, where participants are asked to rate how often they have experienced/witnessed the specific behaviors the last month. A sample item was: “during the last month in your workplace, have you been in a situation where a supervisor or coworker: made demeaning or derogatory remarks about you?” (experienced); and “during the last month in your workplace, have you witnessed a supervisor or coworker: made demeaning or derogatory remarks about others?” (witnessed). Response options ranged from 1 (*never*) to 5 (*most of the time*). Chronbach’s alpha’s (α) for experienced incivility were: 0.88 (t1), 0.90 (t2), and 0.89 (t3). Chronbach’s alpha’s (α) for witnessed incivility were: 0.91 (t1), 0.92 (t2), and 0.92 (t3).

#### Workplace bullying (experienced and witnessed)

Two one-item measures from the Copenhagen Psychosocial Questionnaire [35], were used to measure experienced and witnessed bullying. The Swedish version of the measures was used [36]. The questions were preceded by a definition of bullying: “Bullying means that a person repeatedly is exposed to unpleasant or degrading treatment, and that the person finds it difficult to defend himself or herself against it”. The questions that followed were phrased “Have you been exposed to bullying at your workplace during the last 12 months?”, and “Have you seen someone else be exposed to bullying at your workplace during the last 12 months?”. Response options were: No; Yes a few times; Yes, monthly; Yes, weekly; and Yes, daily. Due to the limited variance (σ^2^ ranged from 0.16 to 0.38) in these measures, we chose to dichotomize the two questions’ response options into 0 (*no*) and 1 (*yes*) responses, where all ratings beside from the “no” response were coded as 1. By doing this, the measures reflect whether or not the individual had been exposed to, or had witnessed, workplace bullying.

#### Psychological well-being

To measure psychological well-being, the WHO-5 Well-Being Index [37] was used. The scale consists of 5 aggregated items. A Swedish version of the scale was used [38]. A sample item was: “I have felt cheerful in good spirits”, with response options ranging from 1 (*never*) to 6 (*all of the time*). Chronbach’s alpha’s (α) were 0.87 (t1), 0.86 (t2), and 0.88 (t3).

### Ethical considerations

The study protocol was approved by the regional ethical review board in Lund, Sweden (dnr 2016/926). A consent form was presented to the participants at each measurement occasion. The consent form contained information stating that participation in the study was voluntary, that responses would be treated confidentially, and that raw data would remain with the research group and not be shared with the union organization. Participants gave active consent in order to proceed to the study.

For transparency, it should be noted that one previous study with different aims and hypotheses has been published on the same data set used in the present study [33]. That study reports on psychosocial factors in the longitudinal relationship between witnessed incivility, instigated incivility, and well-being, which is outside the scope of the present study.

### Statistical analyses

To test the study hypotheses, we selected individuals who had complete data for the t1 and t2 survey, as well as individuals with complete data for the t2 and t3 surveys. We then conducted hierarchical logistic regression analyses in order to investigate whether experienced or witnessed workplace incivility significantly predicted experienced or witnessed workplace bullying over time. Further, we conducted hierarchical linear regression analyses to investigate whether experienced incivility predicted well-being over time when controlling for workplace bullying. To tease out the unique contribution of each factor, both experienced and witnessed forms of incivility and bullying were included in all models. This is necessary as experienced and witnessed behavior showed very large correlations at all time points.

All hierarchical models consisted of two steps. In the first step, prior levels of experienced and witnessed bullying were entered into the equation (t1 variables predicting the t2 outcome, and t2 variables predicting the t3 outcome, respectively). In the next step, the incivility variables were entered into the model to assess whether they significantly contributed to the prediction of the dependent variable, when the variance from workplace bullying over time had already been accounted for. By doing so, we account for the autocorrelation of workplace bullying when testing H1 and H2. In addition, we control for both the possible underlying effect of workplace bullying in the relationship between workplace incivility and well-being, and the autocorrelation of well-being, when testing H3. As no demographic variables were significantly related to any of the dependent variables over time, we did not include them as covariates in the models.

Data were missing completely at random, as suggested by Little’s MCAR test, that was not significant, χ^2^(42) = 48.33, *p* = 0.232. Listwise deletion results in unbiased parameter estimates and standard errors when data are missing completely at random (MCAR) [39]. Although it should be noted that researchers recently have suggested that multiple imputation is advantageous over listwise deletion under conditions of MCAR because it can utilize more of the sample information [40], both listwise deletion and multiple imputation give unbiased results under MCAR [39, 40]. In this case, we used listwise deletion.

## Results

### Descriptive statistics

Table 1 shows descriptive statistics, and Pearson’s correlations of the study variables at all three time points.

**Table 1:** Descriptive statistics (N = 468–489) and Pearson’s correlations (N = 159–489) of observed study variables over the three time points

Variable	1	2	3	4	5	6	7	8	9	10	11	12	13	14	15
*Time 1*															
1. EI															
2. WI	0.75***														
3. EB	0.52***	0.28***													
4. WB	0.43***	0.52***	0.41***												
5. WeB	− 0.37***	− 0.29***	− 0.21***	− 0.14**											
*Time 2*															
6. EI	0.81***	0.65***	0.33***	0.47***	− 0.23										
7. WI	0.67***	0.72***	0.13	0.42***	− 0.17	0.75***									
8. EB	0.55***	0.37***	0.52***	0.37***	− 0.13	0.58***	0.41***								
9. WB	0.40***	0.43***	0.33***	0.44***	− 0.13	0.44***	0.50***	0.37***							
10. WeB	− 0.30***	− 0.15*	− 0.12	− 0.03	0.66***	− 0.37***	− 0.24***	− 0.23***	− 0.14						
*Time 3*															
11. EI	0.58***	0.49***	0.44***	0.26**	− 0.16	0.71***	0.57***	0.49***	0.35***	− 0.27***					
12. WI	0.59***	0.59***	0.32***	0.34***	− 0.18*	0.60***	0.66***	0.28***	0.40***	− 0.25	0.78***				
13. EB	0.37***	0.30***	0.37***	0.34***	− 0.12	0.49***	0.29***	0.65***	0.39***	− 0.14	0.54***	0.42***			
14. WB	0.38***	0.38***	0.19*	0.37***	− 0.11	0.47***	0.40***	0.44***	0.47***	− 0.16*	0.49***	0.58***	0.52***		
15. WeB	− 0.20*	− 0.12	0.05	0.10	0.57***	− 0.26***	− 0.20	− 0.14	− 0.14	0.72***	− 0.31***	− 0.23***	− 0.19***	− 0.10*	–
*Descriptives*															
*M*	1.49	1.67	0.09	0.18	3.93	1.54	1.65	0.09	0.15	3.92	1.51	1.65	0.10	0.16	3.86
*SD*	0.59	0.70	0.30	0.38	0.98	0.64	0.67	0.29	0.35	0.98	0.59	0.66	0.29	0.37	1.00
*N*	489	488	486	489	487	471	471	468	469	471	473	473	472	473	473

*EI* experienced incivility, *WI* witnessed incivility, *EB* experienced bullying, *WB* witnessed bullying, *WeB* well-being

*p < 0.05, **p < 0.01, ***p < 0.001

At each measurement occasion 9.9% (t1), 9.4% (t2), and 9.5% (t3), reported having experienced workplace bullying. In addition, 17.8% (t1), 14.5% (t2), and 16.5% (t3), of the participants reported having witnessed workplace bullying.

### Dropout analysis

In order to investigate whether study dropout was associated with any of the variables, we conducted a series of independent t-tests (for scales) and χ^2^ tests for independence (for binary variables) between those that did not answer a follow up-survey (dropout group, *N* = 431) and those that answered at least two surveys (retained in the study, *N* = 341). We define dropouts as participants that only partook in the t1 or t2 survey, but did not participate in a follow-up measure. Table 2 shows the results from the independent t-tests and χ^2^ tests for independence, including means and standard deviations (for scales) as well as cell counts (for binary variables), test statistics, *p* values, 95% confidence intervals, and effect sizes. There were no significant differences between the groups on experienced incivility, witnessed incivility, experienced bullying, witnessed bullying, or well-being at either of the two time points.

**Table 2:** Dropout analysis with results from independent t-tests and χ^2^-tests for independence comparing study dropouts (completing only one survey at t1 or t2) to those retained (completing at least two surveys), and full adherence participants (completing all three surveys) to partial/non-adherence participants (completing one or two surveys)

	Dropout group (n = 431)		Retention group (n = 341)						
	*M*	*SD*	*M*	*SD*	*t* statistic	*p* Value	Mean difference	95% CI	Cohen’s *d*^a^
EI	1.52	0.66	1.47	0.52	− 0.91	0.361	− 0.05	− 0.16 to 0.06	− 0.08
WI	1.71	0.76	1.64	0.63	− 1.13	0.260	− 0.07	− 0.20 to 0.05	− 0.10
WeB	3.96	0.96	3.90	1.01	− 0.76	0.450	− 0.07	− 0.24 to 0.11	− 0.07
EI.t2	1.59	0.68	1.51	0.60	− 1.38	0.169	− 0.08	− 0.20 to 0.04	− 0.13
WI.t2	1.68	0.70	1.63	0.65	− 0.74	0.460	− 0.05	− 0.17 to 0.08	− 0.07
WeB.t2	4.02	0.98	3.85	0.98	− 1.86	0.064	− 0.17	− 0.35 to 0.01	− 0.17

	Not exposed	Exposed	Not exposed	Exposed	χ^2^ statistic	*p* Value	Difference in proportions	95% CI	Cramer’s *V*
EB	213	19	224	29	1.45	0.287	− 0.03	− 0.09 to 0.02	0.05
WB	189	45	213	41	0.80	0.406	0.03	− 0.04 to 0.10	0.04
EB.t2	177	17	245	27	0.18	0.749	− 0.01	− 0.07 to 0.04	0.02
WB.t2	166	30	234	37	0.25	0.689	0.02	− 0.05 to 0.08	0.02

	Partial/non-adherence (n = 905)		Full adherence group (n = 100)						
	*M*	*SD*	*M*	*SD*	*t* statistic	*p* Value	Mean difference	95% CI	Cohen’s *d*
EI	1.52	0.62	1.37	0.47	2.56*	0.011	0.15	0.02 to 0.28	0.25
WI	1.72	0.72	1.49	0.57	3.42***	< .001	0.23	0.10 to 0.36	0.33
WeB	3.93	0.97	3.92	1.04	0.10	0.920	0.01	− 0.21 to 0.23	0.01
EI.t2	1.58	0.67	1.39	0.49	3.17**	0.002	0.19	0.07 to 0.31	0.30
WI.t2	1.69	0.70	1.50	0.55	2.93**	0.004	0.19	0.06 to 0.32	0.29
WeB.t2	3.93	0.98	3.91	1.00	0.11	0.456	0.01	− 0.21 to 0.23	0.01

	Not exposed	Exposed	Not exposed	Exposed	χ^2^ statistic	*p* Value	Difference in proportions	95% CI	Cramer’s *V*
EB	355	32	83	16	5.52*	0.024	0.08	0.01 to 0.16	0.11
WB	315	74	87	13	1.97	0.188	− 0.06	− 0.14 to 0.02	0.06
EB.t2	333	35	91	9	0.02	1.00	− 0.01	− 0.07 to 0.06	0.01
WB.t2	306	64	95	4	11.07***	< .001	− 0.13	− 0.19 to − 0.08	0.15

*EI* experienced incivility, *WI* witnessed incivility, *EB* experienced bullying, *WB* witnessed bullying, *WeB* well-being

^a^The Cohen’s *d* values are negative as the coding was 0 = retention group, 1 = dropout group. It does not change the interpretation of magnitude of *d*

*p < 0.05, **p < 0.01, ***p < 0.001

However, as can be seen in Table 2, when comparing individuals that participated in all three waves (full adherence) to the full sample, we found that the adherence sample had significantly lower ratings of experienced incivility (*M* = 1.37 compared to *M* = 1.52 at t1, *p* = 0.011, and *M* = 1.39, compared to *M* = 1.58 at t2, *p* = 0.002). The effect sizes were small [41], as Cohen’s *d* = 0.25 at time 1, and *d* = 0.30 at time 2. In addition, the full adherence subsample had significantly lower ratings of witnessed incivility at t1 (*M* = 1.49, compared to *M* = 1.72, *p* = 0.001) and at t2 (*M* = 1.50, compared to *M* = 1.69, *p* = 0.004). Again, with small effect sizes [41], Cohen’s *d* = 0.33 at time 1, and 0.29 at time 2. Lastly, the full adherence sample had a significantly higher amount of experienced bullying at t1, χ^2^(1) = 5.52, *p* = 0.019, Cramer’s *V* = 0.11, and lower amount of witnessed bullying at t2, χ^2^(1) = 11.07, *p* = 0.001, Cramer’s *V* = 0.15, than those that completed one or two surveys. The effect sizes, measured by Cramer’s *V*, were small [41]. This could indicate that individuals with higher exposure to workplace mistreatment were more prone to drop out of the study, although the patterns were only consistent for the incivility variables.

### Hypotheses testing

To test hypothesis 1, if experienced workplace incivility was significantly positively related to self-reported bullying exposure over time (from t1 to t2, and from t2 to t3), we conducted two hierarchical regression analyses. In the first model, experienced bullying at t1 was a significant predictor of experienced bullying at t2, OR = 14.88, (95% CI 3.77–58.72), *p* < 0.001 in step 1. Witnessed bullying did not contribute significantly to the prediction. In the next step, the two incivility variables were added. Experienced incivility at t1 emerged as a significant predictor of experienced bullying at t2, OR = 14.27, (95% CI 2.55–79.98), *p* < 0.01, above and beyond the contribution of the bullying variables. Witnessed incivility did not predict experienced bullying at t2. In the next model, experienced bullying at t2 was a significant predictor of experienced bullying at t3 OR = 38.09, (95% CI 9.39–154.44), *p* < 0.001. In addition, witnessed bullying at t2 significantly predicted experienced bullying at t3, OR = 5.18, (95% CI 1.13–23.81), *p* < 0.05. When adding the incivility variables in step 2, it was again found that experienced incivility at t2 predicted experienced bullying at t3, OR = 6.81, (95% CI 1.55–29.99), *p* < 0.05, but this time with a slightly weaker effect. Overall, the two models showed consistent support for hypothesis 1. The odds ratios were in all cases large than 5, which is suggested to indicate a large effect size [42]. The odds ratios and confidence intervals of all parameters for these analyses are shown in Table 3.

**Table 3:** Odds ratios and confidence intervals of variables predicting experienced and witnessed bullying at t2 (time 1 predictors) and t3 (time 2 predictors) respectively

	Predictor t1	Predictor t2	Time 2				Time 3			
			Experienced Bullying t2 (n = 187)		Witnessed Bullying t2 (n = 186)		Experienced Bullying t3 (n = 180)		Witnessed Bullying t3 (n = 180)	
			OR	95% CI	OR	95% CI	OR	95% CI	OR	95% CI
Step 1	EB t1	EB t2	14.88***	3.77–58.72	2.42	0.65–9.01	38.09***	9.39–154.44	7.78**	2.21–27.42
	WB t1	WB t2	2.87	0.72–11.43	10.29***	3.07–34.51	5.18*	1.13–23.81	10.84***	3.43–34.28
Step 2	EB t1	EB t2	14.61**	2.38–89.55	4.55	0.87–23.89	19.02***	4.37–82.79	4.33*	1.12–16.76
	WB t1	WB t2	1.24	0.20–7.94	3.04	0.73–12.67	4.83	0.92–25.29	5.70**	1.60–20.30
	EI t1	EI t2	14.27**	2.55–79.98	0.99	0.23–4.34	6.81*	1.55–29.99	2.67	0.79–8.98
	WI t1	WI t2	0.906	0.19–4.42	4.50*	1.22–16.65	0.46	0.11–1.85	1.50	0.53–4.25

*EB* experienced bullying, *WB* witnessed bullying, *EI* experienced incivility, *WI* witnessed incivility

^*^p < 0.05, **p < 0.01, ***p < 0.001

To test hypothesis 2, if witnessed workplace incivility was significantly positively related to self-reports of witnessed bullying over time (from t1 to t2, and from t2 to t3), the same procedure was followed, but witnessed bullying was now the dependent variable. In the first model, witnessed bullying at t1 significantly predicted witnessed bullying at t2, OR = 10.29, (95% CI 3.07–34.51), *p* < 0.001. Experienced bullying at t1 did not significantly predict witnessed bullying at t2. In the next step, when adding the incivility variables, witnessed incivility at t1 significantly predicted witnessed bullying at t2, in support of hypothesis 2. The OR = 4.50 (95% CI 1.22‒16.65), *p* < 0.05, suggested a medium effect size [42]. However, when testing the associations between t2 and t3, only experienced and witnessed bullying at t2 significantly predicted witnessed bullying at t3, the incivility variables did not contribute significantly to the model. Hypothesis 2 was therefore only partly supported. See Table 3 for the odds ratios and confidence intervals of the parameters for each model.

To test hypothesis 3, if experienced workplace incivility was significantly negatively related to psychological well-being over time, above and beyond the contribution of workplace bullying in the same model (from t1 to t2, and t2 to t3), two hierarchical linear regression models were estimated. In the first, well-being at t2 was the dependent variable, in the second model, well-being at t3 was the dependent variable. To control for the autocorrelation of well-being over time, well-being from the previous time point was entered into the model together with the predictors. The autocorrelations showed strong significant relationships consistently over the models, βs were 0.62, (95% CI 0.49–0.70), *p* < 0.001, and 0.72, (95% CI 0.66–0.90), *p* < 0.001. Interestingly, neither experienced nor witnessed bullying appeared to significantly relate to well-being over time at either of the time points. The only significant association, beside the autocorrelation, was experienced incivility at t1 which negatively predicted well-being at t2, β = 0.28, (95% CI − 0.88 to − 0.18), *p* < 0.01, above and beyond the contribution of workplace bullying. The partial eta squared (η^2^_p_) for this parameter was 0.05, indicating a small effect size [43]. But this finding was not replicated for the t2–t3 estimates. Hypothesis 3 was therefore only partly supported. Parameter estimates, confidence intervals and effect sizes of parameters in the linear models are shown in Table 4.

**Table 4:** Standardized beta coefficients, confidence intervals, and partial eta squares of variables predicting well-being at t2 (time 1 predictors) and t3 (time 2 predictors) respectively

	Predictor t1	Predictor t2	Time 2			Time 3		
			Well-Being t2 (n = 186)			Well-Being t3 (n = 180)		
			β	95% CI	η^2^_p_	β	95% CI	η^2^_p_
Step 1	Well-Being t1	Well-Being t2	0.66***	0.52–0.74	0.43	0.72***	0.66–0.89	0.50
	EB t1	EB t2	− 0.07	− 0.60–0.20	0.01	− 0.01	− 0.41–0.39	0.00
	WB t1	WB t2	0.06	− 0.18–0.52	0.01	0.01	− 0.34–0.41	0.00
Step 2	Well-Being t1	Well-Being t2	0.62***	0.49–0.70	0.40	0.72***	0.66–0.90	0.47
	EB t1	EB t2	− 0.01	− 0.43–0.41	0.00	− 0.01	− 0.46–0.40	0.00
	WB t1	WB t2	0.09	− 0.14–0.62	0.01	0.01	− 0.38–0.43	0.00
	EI t1	EI t2	− 0.28**	− 0.88 to − 0.18	0.05	0.03	− 0.27–0.38	0.00
	WI t1	WI t2	0.13	− 0.09–0.49	0.01	− 0.02	− 0.29–0.24	0.00

*EB* experienced bullying, *WB* witnessed bullying, *EI* experienced incivility, *WI* witnessed incivility

**p < 0.01, ***p < 0.001

## Discussion

The aim of the present study was to explore whether workplace incivility could be a risk factor for workplace bullying. An additional aim was to explore whether workplace incivility is associated with psychological well-being above and beyond the influence of workplace bullying on well-being. We investigated whether experienced and witnessed workplace incivility significantly contributed to the prediction of experienced or witnessed workplace bullying, or psychological well-being, over time. Specifically, we investigated the contribution of the incivility variables when controlling for prior levels of workplace bullying. We found that the likelihood of being targeted by workplace bullying was higher for those who had previously experienced incivility, even when taking previous bullying exposure into account. In other words, experienced workplace incivility appears to be a risk factor for future bullying exposure. There was also partial support indicating that witnessing incivility could be a risk factor for witnessing bullying at a later time point. Collectively, this suggests that workplace incivility can be a precursor to bullying in the workplace. Moreover, the results suggest that experienced workplace incivility negatively impacts psychological well-being over time, even when accounting for the effect of experienced and witnessed workplace bullying on well-being. However, this result did not replicate at the subsequent measurement occasion.

The results in support of H1, and in partial support of H2, indicate that workplace incivility can be a risk factor for future bullying. This is in line with the escalation models that have been proposed in previous research [11, 16, 20–22]. The results pertaining to experienced incivility and experienced bullying were most consistently supported. The results could possibly be interpreted in line with the theorization that workplace mistreatment may start with more subtle, ambiguous mistreatment, that over time becomes more systematic and develops into more severe transgressions [12]. Previous research has shown that earlier stages of victimization, akin to experiencing incivility, are characterized mostly by work-related negative acts, whereas more severe cases of systematic bullying were associated with an increased amount of person-oriented negative acts [21]. This falls in line with Leymann’s process-model of bullying where an initial work-related conflict subsequently escalates to person-oriented harassment [20]. The findings of the present study could indicate a similar process, where experienced incivility takes place early in a victimization process, subsequently developing into more overt harassment when repeated over time. Another possible explanation for the findings is that there could be a common underlying factor that causes both workplace incivility and workplace bullying to co-occur (the third variable problem [44]). For instance, other factors in the work environment could increase the risk for both incivility and bullying, resulting in the constructs being highly correlated. If this is the case, it may not be incivility specifically, but instead some other element that is the main risk factor for workplace bullying. Nevertheless, from a practical perspective, the presence of workplace incivility could then still indicate a risk for workplace bullying, and be a signal that there is something wrong in the work environment.

H2 regarding witnessed incivility was only partly supported in the present study. Andersson and Pearson [12] described that there could be a risk that norms for civility erode over time, if incivility in the workplace is normalized. In that way, it is possible that the increased risk of witnessing bullying after having previously reported witnessed incivility, reflects a deteriorating social culture in the workplaces where incivility was taking place. Norms for respect may have been increasingly hollowed, resulting in abusive behavior becoming normalized and more frequently occurring in the workplace. As incivility has been suggested to run the risk of spreading and becoming established in the workplace culture over time [17, 19], it is possible that the findings of the present study reflect that incivility can act as a fertilizer for more severe mistreatment. Nevertheless, the results were not replicated over measurement occasions. Witnessed incivility therefore appears to be a less pronounced risk factor for bullying, compared to experienced incivility.

The results partially supported H3, concerning well-being. This is in line with the findings of Yao et al. [31] suggesting that workplace incivility uniquely contributes to the prediction of lower levels of well-being, above and beyond the variance explained by workplace bullying. In other words, mistreatment of low intensity may also be harmful to employee well-being. Possibly through the mechanism described by Cortina et al. [28], that daily hassles result in allostatic load, which over time taxes the individual’s resources, resulting in strain-related outcomes. However, it was surprising that experienced or witnessed workplace bullying did not significantly predict well-being over time, although there were significant cross-sectional correlations between the constructs at each time point. One possible explanation for this could be that victims of bullying may have been subjected to mistreatment over a longer period of time, resulting in steady, but lower, levels of well-being throughout the surveyed period. A possible causal effect of bullying on well-being may then have been exerted at a previous time, before any of the survey measurements, resulting in failure to capture any variation in well-being due to bullying. This is consistent with significant negative cross-sectional correlations at each time point, which means that individuals exposed to bullying had significantly lower mean ratings of well-being than those not exposed to bullying, consistently over the three waves. The consequences of workplace incivility, on the other hand, may appear over time due to larger fluctuations and variation of low intensity behavior [31]. Nevertheless, this relationship was not replicated at the second time point, providing only weak support for longitudinal effects of experienced incivility on psychological well-being.

### Theoretical and methodological contributions

The present study makes several important theoretical and methodological contributions. Specifically, we empirically test a prerequisite for the escalation models of workplace bullying, namely whether a form of low intensity mistreatment can be a risk factor for more severe subsequent mistreatment. This contributes to our knowledge about how workplace mistreatment can evolve and develop over time in the workplace. Considering that few studies have attempted to study whether workplace mistreatment can become increasingly intense over time, this knowledge provides novel insight into the bullying process, as well as highlights the potential severity of a low intensity stressor such as workplace incivility. In addition, the present study provides a methodological contribution as well. By testing whether there is a direct relationship between experienced incivility and well-being over time, when accounting for the variance explained by workplace bullying, we contribute knowledge about the unique impact of incivility on well-being. This validates the conclusions drawn in previous studies, where relationships between experienced incivility and well-being were found (e.g. [24–26]). This demonstrates that incivility uniquely can contribute to the prediction of lower well-being when more severe mistreatment has been partialled out. Furthermore, the present study also makes a methodological contribution by testing the relationship between experienced incivility and well-being over a longer time lag, showing that there is partial support for a relationship over longer periods of time, extending previous cross-sectional findings.

### Practical implications

The findings of the present study have some practical implications. For one, if workplace incivility can constitute a risk factor for future bullying, it stresses the importance of addressing workplace mistreatment at an early stage. Specifically, secondary interventions attempting to target incivility could also function as a primary intervention, and work preventatively against workplace bullying. The utility of incivility interventions, such as the Civility, Respect and Engagement in the Workforce (CREW; [45]), may therefore be greater than previously known. From a managerial point of view, these findings also highlight the importance of acting against incivility immediately, in order to avoid conflict escalation, and the subsequent risk of bullying. Furthermore, as we found a significant relationship between experienced incivility and well-being over time, when controlling for bullying, it is important in its own regard to address incivility when it occurs—in order to avoid negative health effects for employees. Organizational policy documents, managerial practices, and workplace interventions could be synchronized in order to address the potential negative ramifications of low intensity mistreatment. Specifically, by acknowledging the importance of not allowing unacceptable behavior to pass because it did not seem overtly severe, taking immediate action, and recognizing the long term benefits of a respectful social environment for reducing occupational hazards.

### Limitations and future research

The present study has several strengths, such as the longitudinal design, validated measurement instruments, and a thorough dropout analysis. Nevertheless, there are several limitations that should be accounted for. First and foremost, a one-item measure was used to measure experienced and witnessed workplace bullying in the study. This is referred to as the self-labelling method of measuring workplace bullying [46]. This method can be problematic, as research has shown that respondents have a tendency to underestimate bullying when responding to one-item measures [46]. On the other hand, it would not be possible to use a rating scale such as the NAQ-R to measure bullying in the present study, due to the large content overlap with the WIS. The scales would be too highly correlated to discern any construct specific variance, without actually measuring key features of each construct, such as intentionality, persistence over time, and power differences. By using the self-labelling measure, we were able to capture specific features of workplace bullying, such as systematic exposure and the power differential between victim and perpetrator. In this case, we argue that the benefits of the self-labelling method outweighs its limitations. Nevertheless, future studies with more refined measures for both workplace incivility and workplace bullying are needed in order to better understand their interrelation.

On a similar note, it was not possible to conduct a complete case analysis (same subjects at all three time points) of the hypotheses, as we only had *N* = 99 respondents left with complete data for the variables at all three time points when accounting for individuals that had shifted workplace over the course of the study. When running the models with such a small sample, collinearity issues prevented us from obtaining parameter estimates and confidence intervals for the factors. Instead, the test over the second time lag could be considered a replication of the test over the first time lag, which contained about 99 of the same individuals, and a few new participants. It should also be noted that the confidence intervals were large in the models that were estimated, particularly concerning the bullying factors. This is likely due to the small sample size and the low occurrence of workplace bullying, which could result in a higher level of uncertainty of estimates. Although the present study provides a first indication that low intensity mistreatment is associated with high intensity mistreatment over time, and that workplace incivility incrementally predicts psychological well-being when controlling for workplace bullying, more studies are needed to explore these effects with increased precision. This could possibly be remedied by replications with larger samples in future studies, again with more refined measures of each construct. Preferably, future replication studies could be pre-registered, including an a priori power analysis, to further increase credibility and transparency.

Moreover, the dropout analysis demonstrated that there were some significant differences between the full adherence group and the rest of the sample. Of particular importance, it was shown that individuals subjected to mistreatment were more prone to drop out of the study. This suggests that our findings may not be representative, and that the parameter estimates may be under or overestimated. Most likely, the association may be underestimated due to restriction of range, which has a tendency to reduce estimates [47]. In future studies, the risk of workplace bullying due to workplace incivility may be better estimated with higher retention rates. Additionally, different time lags can be employed to (1) investigate how this influences attrition rates, and (2) explore over which time frames workplace incivility enhance the risk for bullying. This can be informative for understanding how long it can take for workplace bullying to potentially develop after exposure to incivility. Furthermore, due to the low response rate, the generalizability of the findings to the population of Swedish engineers is limited. Although the study sample was drawn at random from the member registry of a large Swedish union for engineers, the respondents may not be representative of the full population of Swedish engineers. In addition, the findings may not be generalizable to other populations. Future replications could aim to explore these findings in other work sectors, using stratified sampling to obtain a sample representative of the target population.

Lastly, it is possible that an unmeasured third variable, common to both workplace incivility and workplace bullying, could explain the relationships found in the present study. The third variable problem is always an issue in observational research that limits causal inferences [44]. Nevertheless, the findings of the present study are important, as workplace incivility still could indicate that there is a risk for more severe mistreatment, regardless of the underlying causes. Future studies could attempt to explore antecedents common to both workplace incivility and workplace bullying, in order to delineate whether workplace incivility is at risk of escalating into more severe mistreatment, or whether there are other factors in the work environment that create a heightened risk for multiple types of mistreatment. In that case the focus should be on when and why it develops into bullying.

### Conclusions

The results demonstrated that individuals that have experienced workplace incivility are at heightened risk of being exposed to workplace bullying. In addition, having witnessed incivility was partly associated with increased risk of subsequently witnessing bullying. Taken together, this suggests that workplace incivility can be a risk factor for future bullying. The results also showed that experienced workplace incivility had a negative effect on psychological well-being over time, even when accounting for the impact of workplace bullying on well-being. This underlines that it is important to address workplace incivility not only because it is a risk factor for future severe mistreatment, but also due to its unique negative impact on psychological well-being.

## Data Availability

The data that support the findings of this study are available on request (in a de-identified manner) from the corresponding author. The data are not publicly available due to privacy or ethical restrictions.

## Abbreviations

EU: European Union
CI: Confidence interval
CREW: Civility, Respect and Engagement in the Workforce
H1: Hypothesis 1
H2: Hypothesis 2
H3: Hypothesis 3
MCAR: Missing completely at random
NAQ-R: Negative Acts Questionnaire-Revised
OR: Odds ratio
SD: Standard deviation
t1: Time 1
t2: Time 2
t3: Time 3
WHO: World Health Organization
WIS: Workplace Incivility Scale
